# The addition of chemotherapy to adjuvant radiation is associated with inferior survival outcomes in intermediate‐risk HPV‐negative HNSCC

**DOI:** 10.1002/cam4.3883

**Published:** 2021-05-02

**Authors:** Jason Tasoulas, Nicholas R. Lenze, Douglas Farquhar, Travis P. Schrank, Colette Shen, M. Ali Shazib, Bart Singer, Shetal Patel, Juneko E. Grilley Olson, David N. Hayes, Margaret L. Gulley, Bhishamjit S. Chera, Trevor Hackman, Andrew F. Olshan, Jared Weiss, Siddharth Sheth

**Affiliations:** ^1^ Department of Otolaryngology‐Head and Neck Surgery The University of North Carolina at Chapel Hill Chapel Hill NC USA; ^2^ Department of Radiation Oncology The University of North Carolina at Chapel Hill Chapel Hill NC USA; ^3^ Division of Diagnostic Sciences Adams School of Dentistry The University of North Carolina at Chapel Hill Chapel Hill NC USA; ^4^ Department of Pathology The University of North Carolina at Chapel Hill Chapel Hill NC USA; ^5^ Division of Medical Oncology Department of Medicine The University of North Carolina at Chapel Hill Chapel Hill NC USA; ^6^ Division of Hematology‐Oncology Department of Medicine University of Tennessee Health Science Center Memphis TN USA; ^7^ Department of Epidemiology Gillings School of Global Public Health The University of North Carolina at Chapel Hill Chapel Hill NC USA

**Keywords:** adjuvant radiotherapy, chemotherapy, head and neck cancer, head and neck squamous cell carcinoma, HPV‐negative, risk factors, survival

## Abstract

**Background:**

Only high‐risk tumors with extranodal extension (ENE) and/or positive surgical margins (PSM) benefit from adjuvant therapy (AT) with concurrent chemoradiation (CRT) compared to radiation therapy (RT) in locally advanced head and neck squamous cell carcinoma (HNSCC). Optimal treatment for intermediate‐risk tumors remains controversial. We categorized patients based on their surgical pathologic risk factors and described AT treatment patterns and associated survival outcomes.

**Methods:**

Patients were identified from CHANCE, a population‐based study, and risk was classified based on surgical pathology review. High‐risk patients (n = 204) required ENE and/or PSM. Intermediate‐risk (n = 186) patients had pathological T3/T4 disease, perineural invasion (PNI), lymphovascular invasion (LVI), or positive lymph nodes without ENE. Low‐risk patients (n = 226) had none of these features.

**Results:**

We identified 616 HPV‐negative HNSCC patients who received primary surgical resection with neck dissection. High‐risk patients receiving AT had favorable OS (HR 0.50, *p *= 0.013) which was significantly improved with the addition of chemotherapy compared to RT alone (HR 0.47, *p *= 0.021). When stratified by node status, the survival benefit of AT in high‐risk patients persisted only among those who were node‐positive (HR: 0.17, *p *< 0.0005). On the contrary, intermediate‐risk patients did not benefit from AT (HR: 1.26, *p *= 0.380) and the addition of chemotherapy was associated with significantly worse OS compared to RT (HR: 1.76, *p *= 0.046).

**Conclusion:**

In high‐risk patients, adjuvant chemoradiotherapy improved OS compared to RT alone. The greatest benefit was in node‐positive cases. In intermediate‐risk patients, the addition of chemotherapy to RT increased mortality risk and therefore should only be used cautiously in these patients.

## Graphical abstract

The addition of chemotherapy to adjuvant radiotherapy could improve survival. This is observed only among high‐risk patients. When stratified by nodal status, the benefit exists only in node‐positive patients. In intermediate‐risk patients chemotherapy addition decreases survival. Risk categorization is important for treatment selection and survival outcomes.

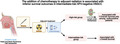

## 1 INTRODUCTION

Head and neck squamous cell carcinoma (HNSCC) is the sixth most common malignancy worldwide.^1^ It constitutes a family of epithelial malignancies including oral cavity, oropharyngeal, and laryngeal squamous cell carcinomas.^2^ Despite improvement in therapeutic options, 5‐year overall survival (OS) has remained relatively unchanged at around 50–60%.^3^, ^4^, ^5^ Historically, TNM stage has been the most important predictor of OS.^5^ However, several reports have identified that clinical (nodal disease in levels IV and V) and pathological factors (extranodal extension, ENE; positive surgical margins, PSM; perineural invasion, PNI, and lymphovascular invasion, LVI) affect prognosis and predict poor locoregional control, worse OS, and worse relapse‐free survival (RFS).^6^, ^7^, ^8^, ^9^ Notably, the 8th AJCC/UICC edition implemented ENE in the nodal (N)‐category of TNM staging.^10^

Therapeutically, primary surgical resection with or without adjuvant therapy remains a standard of care option for patients with HNSCC.^7^ In the phase III EORTC 22391 and RTOG 95–01 trials, HNSCC patients with ENE and/or PSM had improved survival outcomes following adjuvant cisplatin‐based CRT compared to adjuvant radiotherapy (aRT) alone ^11^, ^12^, ^13^, ^14^ and thus has become standard of care treatment practice. Furthermore, adjuvant chemoradiation (aCRT) is suggested for all patients with adverse risk features (ARF), including ENE, PSM, PNI, nodal disease in levels IV and V, LVI).^7^ However, in patients without ARFs, there is no consensus for use in the adjuvant setting. In two National Cancer Database studies of HNSCC patients, the use of aCRT did not confer a survival benefit in patients without positive margins or ENE.^15^, ^16^ One of the studies identified an increasing survival benefit associated with the extent of nodal involvement.^16^ Given the significant adverse events associated with aCRT and the impact on quality of life (QoL), optimizing the selection of adjuvant therapy (AT) is essential to maximize survival and minimize treatment‐associated adverse outcomes.^17^, ^18^

We sought to address the impact of AT on the survival of HPV‐negative HNSCC patients in a large population‐based database from North Carolina. We used an ARF‐based patient classification scheme described by the National Comprehensive Cancer Network (NCCN) and commonly cited literature.^7^, ^19^, ^20^, ^21^ High risk included only patients with ENE and/or PSM. Intermediate‐risk was defined as pathological T3/T4 disease, PNI, LVI, or positive lymph nodes without ENE or PSM. Patients without an ARF were defined as low‐risk.

## 2 METHODS

### 2.1 Study Population

Patients diagnosed with HPV‐negative HNSCC were identified from the Carolina Head and Neck Cancer Epidemiology Study (CHANCE), a population‐based study in North Carolina.^22^ Cases were eligible to participate in CHANCE if they had been diagnosed with a first primary squamous cell carcinoma of the oral cavity, pharynx, or larynx between January 1, 2002, and February 28, 2006; aged 20 to 80 years at diagnosis; and resided in a 46‐county region in central North Carolina. The study was approved by the Institutional Review Board (IRB) of all participating institutions.

For each patient who received primary surgical resection, medical records were retrospectively reviewed and the following data were collected: age, sex, tumor site, surgical treatment category (surgical resection only, surgical resection and aRT, surgical resection and aCRT), HPV tumor status, LVI, PNI, ENE, T stage, and number of positive lymph nodes. HPV tumor status was evaluated using p16 immunohistochemistry and was performed by the International Agency for Research on Cancer (IARC). The full p16 immunohistochemistry protocol has been previously described.^23^ For the purpose of this study, patients with p16‐positive oropharyngeal cancer were excluded, as HPV‐associated HNSCC had a distinct biological behavior and more favorable prognosis. Information on individual behaviors (tobacco and alcohol use) and socioeconomic status (household income, education, and insurance status) was collected through in‐home interviews by trained nurse interviewers during the creation of CHANCE. Race was self‐identified from an interview question.

Staging classification was based on the American Joint Commission on Cancer (AJCC) 7^th^ edition, which was available at the time of data collection.

### 2.2 Statistical Analysis

Descriptive statistics were calculated, and bivariate testing methods included chi‐square and Fisher's exact tests. An alpha criterion of *p* ≤ 0.05 was used for all significance testing. CHANCE data were linked to the National Death Index (NDI) based on name, social security number, date of birth, sex, race, and state of residence to identify deaths through December 31, 2013. Overall survival (OS) was estimated using hazard ratios (HR) and corresponding 95% confidence intervals (CI) obtained via Cox regression models stratified by adverse risk group. Models were examined before and after adjusting for the following covariates: tumor site, age, sex, race, smoking, alcohol, TNM stage, treatment, and pathologic risk group Stata 15.1 (StataCorp LP, College Station, TX) was used for all statistical analyses.

## 3 RESULTS

### 3.1 Baseline Characteristics By Risk Category

We identified 616 HPV‐negative HNSCC patients who received primary surgical resection and were classified as either low‐risk (n = 226), intermediate‐risk (n = 186), or high‐risk (n = 204) based on pathological outcomes (Table 1). The majority of patients were male (72%) and identified as white race (72%). Intermediate‐ and high‐risk patients were more likely to be male, lack medical insurance, have a household income < $20,000, use tobacco, and drink alcohol. Intermediate‐risk and high‐risk patients had lower socioeconomic status compared to patients classified as low‐risk. As expected in an HPV‐negative population, most patients in the cohort had drinking (84%) defined as >1 drink per week and heavy smoking histories (78%) defined as >10 pack‐years.

**TABLE 1:** Demographic characteristics of HPV‐negative HNSCC patients by risk category

	Pathologic risk category								
	Low Risk ( n = 226)		Intermediate Risk (n = 186)		High Risk ( n = 204)		Total ( n = 616)		*p*‐value
	No.	%	No.	%	No.	%	No.	%	
Age									0.281
<50	42	19	43	23	43	21	128	21	
50–65	102	45	92	49	87	43	281	46	
>65	82	36	51	27	74	36	207	34	
Sex									<0.005
Male	142	63	137	74	163	80	442	72	
Female	84	37	49	26	41	20	174	28	
Race									0.062
White	179	79	127	68	140	69	446	72	
Black	43	19	56	30	61	30	160	26	
Other	4	2	3	2	3	1	10	2	
Education									0.001
Less Than High School	63	28	61	33	86	42	210	34	
High School Grad	57	25	60	32	60	29	177	29	
Greater than High School	106	47	65	35	58	28	229	37	
Insurance Category									0.025
Private	81	39	60	33	65	33	206	35	
Medicaid/Medicare	71	34	61	34	78	39	210	36	
None	15	7	34	19	22	11	71	12	
Other	40	19	27	15	34	17	101	17	
Income									0.001
Greater than 50 k	66	31	46	26	50	26	162	28	
20 k–50 k	91	43	47	27	68	35	206	35	
<20 k	56	26	81	47	78	40	215	37	
Smoking History									0.016
<10 Pack Years	63	28	38	21	34	17	135	22	
>10 Pack Years	162	72	146	79	170	83	478	78	
Drinking History									0.018
<1 Drink / Week	47	21	20	11	31	16	98	16	
>1 Drink / Week	176	79	166	89	166	84	508	84	

### 3.2 Pathologic and Treatment Characteristics

The larynx/hypopharynx (35%), oral cavity (27%), and oropharynx (12%) were the most common primary tumor sites. Most patients receiving AT (n = 282, 89%) were classified as intermediate‐ or high‐risk (*p *< 0.0005). Among those, 68% (n = 191) received aRT, whereas 32% (n = 91) received aCRT. In the high‐risk group, 79% (n = 158) had PSM, whereas 86% (n = 59) had ENE. Interestingly, only 26% (n = 53) of high‐risk patients received aCRT whereas 50% (n = 103) received aRT. Among intermediate‐risk patients, 20% (n = 38) received aCRT, 47% (n = 88) received aRT, and 32% (n = 60) had no AT. We describe post‐resection pathologic characteristics in detail according to ARF classification (Table 2).

**TABLE 2:** Post‐resection pathological and treatment characteristics of HNSCC patients by risk category

	Pathologic Risk Category								
	Low risk		Intermediate risk		High risk		Total		
	No.	%	No.	%	No.	%	No.	%	*p*‐value
Tumor Site									<0.0005
Oral cavity	80	35	42	23	45	22	167	27	
Oropharynx	15	7	27	15	34	17	76	12	
Larynx/Hypopharynx	53	23	69	37	94	46	216	35	
NOS	78	35	48	26	31	15	157	25	
Surgical Margin									<0.0005
Negative	186	100	158	100	43	21	387	71	
Positive	0	0	0	0	158	79	158	29	
Extranodal Extension									<0.0005
Negative	5	100	48	100	10	14	63	52	
Positive	0	0	0	0	59	86	59	48	
T‐Stage									<0.0005
Early stage (1–2)	226	100	67	36	133	65	426	69	
Advanced stage (3–4)	0	0	119	64	71	35	190	31	
Lymphovascular Invasion									<0.0005
No	159	95	117	81	145	77	421	84	
Yes	8	5	28	19	43	23	79	16	
N‐Stage									<0.0005
N‐negative	226	100	59	32	104	51	389	63	
N‐positive	0	0	127	68	100	49	227	37	
Surgical Treatment Category									<0.0005
Surgery Only	192	85	60	32	48	24	300	49	
Surgery + RT	33	15	88	47	103	50	224	36	
Surgery + CRT	1	0	38	20	53	26	92	15	

### 3.3 Overall survival by pathologic risk category

**FIGURE 1:**
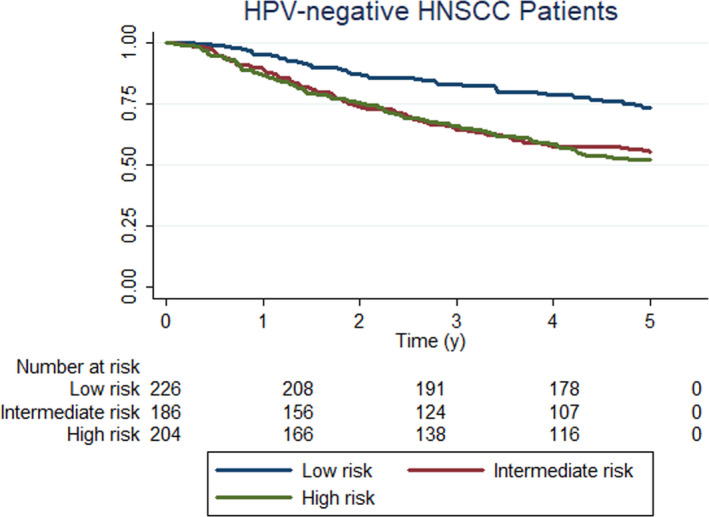
Unadjusted overall survival curves for HPV‐negative HNSCC patients according to pathologic risk category

There were significant differences in overall survival among HPV‐negative HNSCC patients classified by risk. The crude 5‐year OS rate was 73% for low‐risk patients, 55% for intermediate‐risk patients, and 52% for high‐risk patients (Figure 1).

**FIGURE 2:**
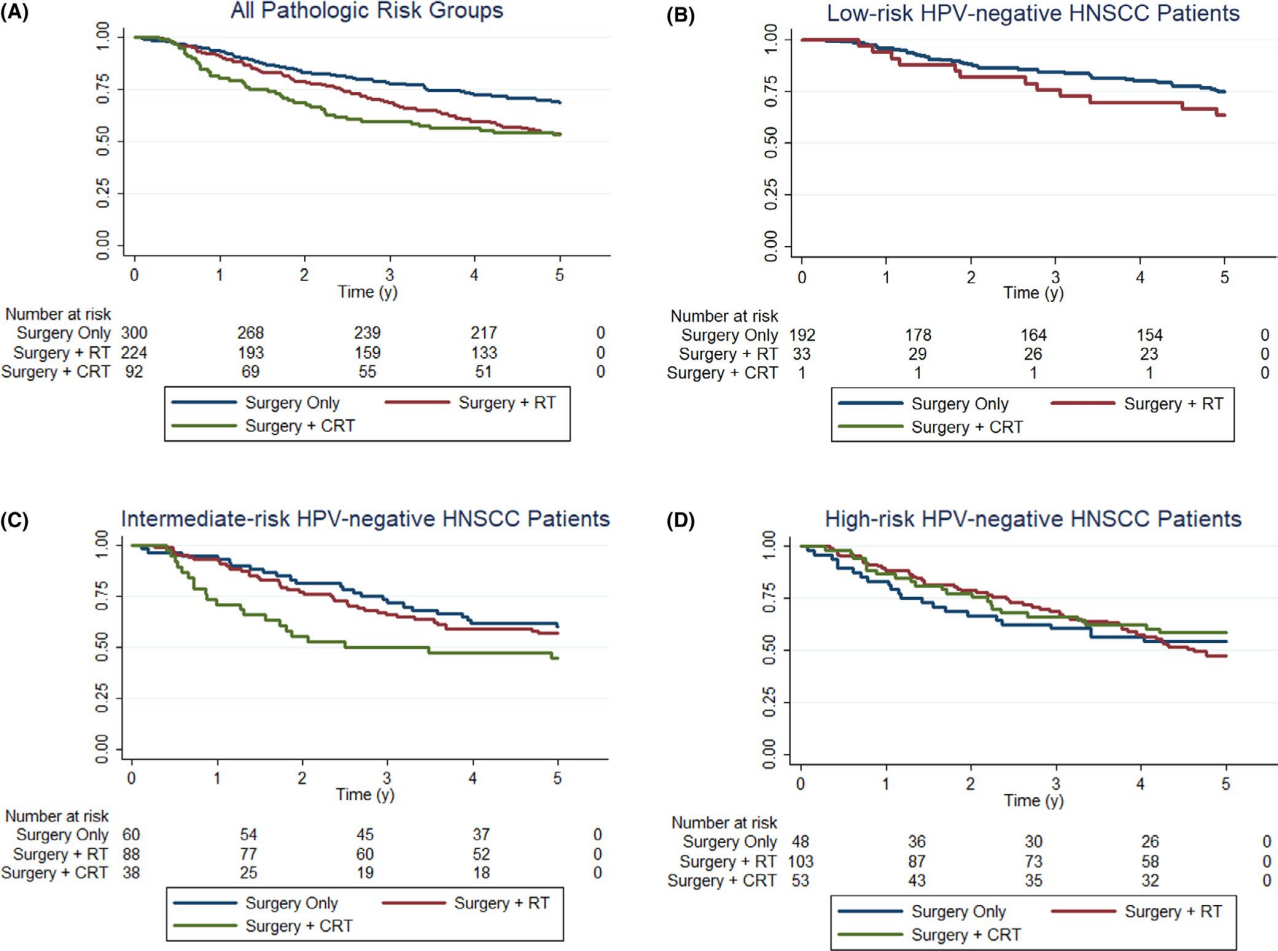
Unadjusted overall survival curves for all HPV‐negative HNSCC patients according to treatment via surgery vs. surgery and radiotherapy vs. surgery and chemoradiotherapy (A), low‐risk HPV‐negative HNSCC patients according to treatment via surgery vs. surgery and radiotherapy vs. surgery and chemoradiotherapy (B), intermediate‐risk HPV‐negative HNSCC patients according to treatment via surgery vs. surgery and radiotherapy vs. surgery and chemoradiotherapy (C), high‐risk HPV‐negative HNSCC patients according to treatment via surgery vs. surgery and radiotherapy vs. surgery and chemoradiotherapy (D)

Patients were stratified by pathological risk category to examine the associations between treatment modality and OS (Table 3 and Figure 2A‐D). All models adjusted for tumor site, age, sex, race, smoking, alcohol, and TNM stage. Among high‐risk patients, those receiving AT had significantly better OS compared to surgery alone (HR 0.50, 95% CI 0.29–0.86, *p *= 0.013). When stratified by adjuvant therapy, aCRT offered significant survival benefit compared to surgery alone (HR 0.30, 95% CI 0.15–0.61, *p *= 0.001) and compared to surgery plus aRT (HR 0.47, 95% CI 0.25 – 0.89, *p *= 0.021). Among intermediate‐risk patients, those receiving AT did not show survival benefit compared to surgery alone (HR 1.26, 95%CI: 0.75–2.10, *p *= 0.380). When stratified by adjuvant therapy, aCRT was associated with worse OS compared to aRT (HR: 1.76, 95%CI 1.01 – 3.05, *p *= 0.046) and surgery alone (HR 1.86, 95%CI 1.00–3.44, *p *= 0.050). Low‐risk patients receiving aRT (HR 2.01, 95% CI 0.95–4.25; *p *= 0.068) had no significant difference in OS compared to surgery alone.

**TABLE 3:** Association of stage and treatment with overall survival in HPV‐negative HNSCC patients

	Low risk		Intermediate risk		High risk	
Cox regression model^a^	HR (95% CI)	*p*‐value	HR (95% CI)	*p*‐value	HR (95% CI)	*p*‐value
TNM Classification						
I	*reference*		—	—	*reference*	
II	1.48 (0.84 – 2.60)	0.173	—	—	1.02 (0.44 – 2.32)	0.970
III	—		*reference*		1.90 (0.75 – 4.83)	0.178
IV	—		1.39 (0.84 – 2.28)	0.198	3.62 (1.97 – 6.65)	<0.0005
Treatment						
Surgery	*reference*		*reference*		*reference*	
Surgery + Radiotherapy	2.01 (0.95 – 4.25)	0.068	1.06 (0.61–1.83)	0.846	0.60 (0.34–1.07)	0.083
Surgery + Chemoradiotherapy	—	—	1.86 (1.00–3.44)	0.050	0.30 (0.15–0.61)	0.001

The numbers of low risk patients receiving surgery + chemoradiotherapy were insufficient for survival analysis.

Abbreviations: CI, Confidence intervals; HR, Hazard ratio.

^a^ Estimates obtained from multivariable Cox regression modeling including terms for tumor site, age, sex, race, smoking, alcohol, TNM stage (AJCC 7th Edition), treatment and pathologic risk group.

Next, we performed a subset analysis for OS stratified by both pathologic risk category and nodal status (positive vs. negative). We found that the survival benefit of AT among high‐risk patients was present only in the node‐positive group (HR 0.17, 95% CI 0.07–0.41, *p *< 0.0005), and this association persisted when evaluating aRT (HR 0.26, 95% CI: 0.10 – 0.67, *p *= 0.006) and aCRT (HR 0.13, 95% CI 0.05 – 0.33, *p *< 0.0005) independently, compared to surgery only (Table S1). The addition of chemotherapy (aCRT) was also associated with improved OS compared to aRT in this group (HR: 0.46, 95%CI: 0.24–0.90, *p *= 0.022). Node stratification did not reveal any significant survival differences for the intermediate‐ and low‐risk patients.

## 4 DISCUSSION

Our findings suggest that intermediate‐risk patients may not benefit from the addition of AT to surgery (*p *= 0.380). Furthermore, the addition of chemotherapy may be associated with an increased risk of mortality compared to aRT (*p *= 0.046) and surgery alone (*p *= 0.050) in this group. This is in contrast to high‐risk patients, who show superior overall survival with AT (*p *= 0.013), which is further improved with the addition of chemotherapy to aRT (*p *= 0.021). This finding is most prominent among high‐risk patients with positive lymph nodes (*p *< 0.0005).

As supported in previous literature, aCRT confers a survival benefit for high‐risk patients, particularly in patients with node‐positive disease based on subset analysis. In contrast, our findings suggest that overtreatment in the adjuvant setting may be detrimental in patients without high‐risk based on pathologic features. The importance of accurately identifying patients that may benefit from adjuvant therapy is critical, given the potentially adverse impact on survival and treatment‐associated morbidity. Thus, adverse risk factors in surgical pathological outcomes should be closely scrutinized for risk stratification and treatment planning.

The lack of survival benefit from treatment intensification in the adjuvant setting for intermediate‐risk patients is supported by evidence from several studies in the current literature. In a large NCDB analysis, Osborn et al. found that the OS survival benefit did not persist for aCRT in patients without positive margins or ENE.^15^ Another study found that aCRT was associated with an OS benefit for non‐oropharyngeal HNSCC patients with T1‐4 N2‐3 disease who were younger than 70 years of age, but not for those who were older than 70 years or had T3‐4 N0‐1 disease.^24^ Among high‐risk patients in our study, we recapitulate the notion that aCRT (HR = .30) is beneficial, as has been consistently shown in other studies. Our study builds upon this prior evidence by demonstrating that nodal status appears to differentially affect treatment response in this group (Table S1).^11^, ^12^, ^13^, ^14^, ^25^

A recent study published by Yan et al. used a similar methodology to investigate the effect of adjuvant chemoradiation therapy on OS for HPV‐negative HNSCC patients receiving primary surgical resection with negative margins from the National Cancer Database.^26^ Patients classified as N2a with a single‐positive lymph node <3 cm and pathologic ENE did not have an OS benefit with CRT relative to adjuvant radiation alone (HR 0.98; 95% CI, 0.74–1.30). These findings were similar to our intermediate‐risk cohort. Despite the presence of pathologic ENE, the overall risk may have been mitigated by the presence of only a single‐positive node and negative surgical margins in this cohort. These findings suggest that even more nuanced pathologic risk scoring systems may be warranted to optimize treatment selection and provide individualized cancer care for patients. This remains a promising area for future research.

The harms of overtreatment in cancer care are often overlooked, and they warrant more discussion. The cytotoxic properties of cisplatin chemotherapy combined with radiation is associated with significant treatment‐related morbidity and mortality, especially in older patients.^27^, ^28^ Acute and chronic effects of combined therapy include mucositis, dysphagia, chronic pain, salivary gland dysfunction, infection, neutropenia, tracheostomy tube dependence, and feeding tube dependence.^27^, ^28^, ^29^ Patients should understand that aCRT in the intermediate‐ or low‐risk setting may diminish functionality and quality of life with potentially little survival benefit, as found in our analysis.

Another main finding of our study is the low percentage of adherence to National Comprehensive Cancer Network (NCCN) guidelines. Patients were enrolled in the CHANCE study between 2002 and 2006. Data from EORTC 22391 and RTOG 95–01 were published in 2004, and it can take years before treatment guidelines are fully integrated into clinical practice. While treatment decisions in this study may not reflect current practice in 2020, investigators who hope to improve survival outcomes via treatment intensification should proceed with caution. This is perhaps most relevant in intermediate‐risk disease, which spans a broad range of clinical and surgical factors. For example, a patient with pathologic T3 disease should only receive adjuvant radiation, whereas a patient with T4 disease, LVSI, and PNI should be considered for aCRT. Unfortunately, our study did not analyze survival outcomes based on a cumulative number of risk factors. Adherence to treatment guidelines remains critical,^30^ as many HNSCC studies report anywhere from 17% to 50% treatment non‐compliance.^30^, ^31^, ^32^ The NCCN guidelines’ category 2A recommendation for aRT (uniform NCCN consensus) in high‐risk patients dates back to the early 2000s.^7^, ^33^ Nevertheless, 24% of high‐risk patients in our study received no AT.

Our study has several limitations. The primary limitation is the retrospective nature of our analysis. As discussed, primary data collection ended in 2006, so treatment patterns may not reflect current practice guidelines. Additionally, pathologic examination of lymph node status (ENE evaluation) was substantially different compared to current practice. Standard evaluation was limited to one histologic section of each node, whereas standardized reporting and College of American Pathologist protocols were not available. Historically, ENE was used to describe a grossly positive or matted node (macroscopic); however, currently, ENE can include a 1 mm focus extending from an equally small metastatic focus (microscopic). Another important limitation is the use of AJCC 7th Edition guidelines, which were available at the time of data collection. To help account for this, we stratified our multivariable analyses by pathological risk category to adjust for tumor characteristics captured in updated staging guidelines. Also, some of the post hoc subgroup analyses were limited by small sample sizes. Another limitation is that HPV‐positive oropharyngeal cases were excluded based on p16‐status alone. While p16 is considered a reliable marker for HPV infection, there is potential for a small number of p16‐positive HPV‐negative cases as shown in other studies.^34^ Finally, the full records for adjuvant therapy received were incomplete. The majority of patients treated with aCRT in this analysis received cisplatin; however, cumulative dosing, and administration schedules (bolus vs. weekly) were not available. Similarly, patients were scheduled to receive the standard of care six weeks of aRT, however, the choice of RT used (conventional vs. intensity modulated) and cumulative dose received was not recorded.

The strengths of our study include a large population‐based sample with complete information on surgical pathologic outcomes. Additionally, our analysis adjusted for potential confounders such as age, sex, race, smoking history, alcohol use, and TNM stage. Given the large sample size and adjustment set, we believe our OS estimates represent true differences in survival by treatment modality and pathologic risk category. These findings help fill an important gap in the current literature and identify areas for future research. Specifically, novel combinatorial therapies such as concurrent radiation therapy plus immunotherapy should be evaluated to improve overall survival outcomes in intermediate‐risk, HPV‐negative patients.

## 5 CONCLUSION

While the use of aRT and aCRT appears to be associated with improved overall survival in high‐risk HPV‐negative HNSCC patients, the same benefit may not be observed in patients with intermediate‐risk disease. In high‐risk patients, node status may be an important parameter of response to treatment. Optimization of risk stratification has the potential to improve treatment selection and survival outcomes for patients with HPV‐negative HNSCC. Additionally, the integration of patient‐reported quality of life measures per treatment modality and risk category may inform decision‐making.

## ETHICAL APPROVAL

The study was approved by the Institutional Review Board (IRB) of the University of North Carolina at Chapel Hill (Approval ID: 17–1220) and all participating institutions.

## CONFLICT OF INTEREST

None.

## AUTHOR CONTRIBUTIONS

Conceptualization: SS, DF; Data Curation: JT, NL, DF; Formal Analysis: JT, NL, DF; Funding Acquisition: AO; Investigation: JT, NL, DF, SS; Methodology: JT, NL, DF, SS; Project Administration: SS; Resources: AO, JW, SS; Software: JT, NL, DF; Supervision: SS; Validation: JT, NL; Visualization: JT, NL; Writing ‐ Original Draft: JT, NL, SS; Writing ‐ Review and Editing: All authors.

## Supporting information

Table S1

## Data Availability

The data that support the findings of this study are available on request from the corresponding author. The data are not publicly available due to privacy or ethical restrictions.
