# Supplementary material for: The addition of chemotherapy to adjuvant radiation is associated with inferior survival outcomes in intermediate‐risk HPV‐negative HNSCC
Source: Cancer Med. 2021 May 2;10(10):3231–9. doi: 10.1002/cam4.3883 (PMC8124130; doi:10.1002/cam4.3883)
Supplement: Supplementary file 1 — Table S1 [file CAM4-10-3231-s001.docx]

| **Supplemental Table 1.** Impact of treatment scheme on OS in relation to pathologic risk group and nodal status, in HPV-negative HNSCC patients | | | | | | | | | | | |
| --- | --- | --- | --- | --- | --- | --- | --- | --- | --- | --- | --- |
| **Pathologic Risk Group** | **Nodal Status** | **Surgery +**  **Radiotherapy**  **(n=241)** | | | **Surgery + Chemoradiotherapy**  **(n=104)** | | | **Adjuvant Therapy**^†^  **(n=345)** | | | |
|  |  | HR | 95% CI | p-value | HR | 95% CI | p-value | HR | 95% CI | p-value |  |
| **Low risk** | N- | 2.01 | 0.95 – 4.25 | 0.068 | - | - | - | 1.81 | 0.86 – 3.80 | 0.115 |  |
|  | N+ | - | - | - | - | - | - | - | - | - |  |
| **Intermediate risk** | N- | 1.93 | 0.52 – 7.07 | 0.324 | 1.93 | 0.45 – 8.22 | 0.372 | 1.93 | 0.62 – 5.98 | 0.255 |  |
|  | N+ | 0.99 | 0.49 – 2.01 | 0.987 | 1.61 | 0.74 – 3.51 | 0.231 | 1.15 | 0.59 – 2.24 | 0.679 |  |
| **High risk** | N- | 0.54 | 0.23 – 1.25 | 0.147 | 3.41 | 0.60 – 19.3 | 0.165 | 0.66 | 0.30 – 1.48 | 0.317 |  |
|  | N+ | 0.26 | 0.10 – 0.67 | 0.006 | 0.13 | 0.05 – 0.33 | <0.001 | 0.17 | 0.07 – 0.41 | <0.001 |  |
| **All groups** | N- | 1.18 | 0.75 – 1.86 | 0.484 | 1.70 | 0.68 – 4.26 | 0.257 | 1.23 | 0.80 – 1.91 | 0.344 |  |
|  | N+ | 0.80 | 0.47 – 1.34 | 0.393 | 0.72 | 0.41 - 1.25 | 0.246 | 0.76 | 0.47 – 1.25 | 0.285 |  |
| OS, overall survival; HR: Hazard ratio; CI: Confidence intervals  ^†^Adjuvant Therapy: Surgery + Radiotherapy or Surgery + Chemoradiotherapy  Estimates obtained from multivariable Cox regression modeling including terms for tumor site, age, sex, race, smoking, alcohol, T- and M- stage, treatment, and pathologic risk group. Surgery alone is the treatment variable reference.  The numbers of low risk patients receiving surgery + chemoradiotherapy and the number of node positive patients receiving surgery + radiotherapy were insufficient for survival analysis. | | | | | | | | | | | |
